# Are within-person Numerical Rating Scale (NRS) ratings of breathlessness ‘on average’ valid in advanced disease for patients and for patients’ informal carers?

**DOI:** 10.1136/bmjresp-2017-000235

**Published:** 2017-10-11

**Authors:** Joshua Wade, Silvia Mendonca, Sara Booth, Gail Ewing, A Carole Gardener, Morag Farquhar

**Affiliations:** 1 School of Clinical Medicine, University of Cambridge, Cambridge, UK; 2 Department of Public Health & Primary Care, University of Cambridge, Cambridge, UK; 3 Department of Oncology, University of Cambridge, Cambridge, UK; 4 Centre for Family Research, University of Cambridge, Cambridge, UK; 5 University of East Anglia, School of Health Sciences, Norwich, UK

**Keywords:** lung cancer, palliative care, perception of asthma/breathlessness, respiratory measurement, emphysema

## Abstract

**Introduction:**

The Numerical Rating Scale (NRS) is frequently used to assess patient-reported breathlessness in both a research and clinical context. A subgroup of patients report average breathlessness as worse than their worst breathlessness in the last 24 hours (paradoxical average). The Peak/End rule describes how the most extreme and current breathlessness influence reported average. This study seeks to highlight the existence of a subpopulation who give ‘paradoxical averages using the NRS, to characterise this group and to investigate the explanatory relevance of the ‘Peak/End’ rule.

**Methods:**

Data were collected within mixed method face-to-face interviews for three studies: the Living with Breathlessness Study and the two subprotocols of the Breathlessness Intervention Service phase III randomised controlled trial. Key variables from the three datasets were pooled (n=561), and cases where participants reported a paradoxical average (n=45) were identified. These were compared with non-cases and interview transcripts interrogated. NRS ratings of average breathlessness were assessed for fit to Peak/End rule.

**Results:**

Patients in the paradoxical average group had higher Chronic Respiratory Questionnaire physical domain scores on average p=0.042). Peak/End rule analysis showed high positive correlation (Spearman’s rho=0.756, p<0.001).

**Conclusions:**

The NRS requires further standardisation with reporting of question order and construction of scale used to enable informed interpretation. The application of the Peak/End rule demonstrates fallibility of NRS-Average as a construct as it is affected by current breathlessness. Measurement of breathlessness is important for both clinical management and research, but standardisation and transparency are required for meaningful results.

## Introduction

Dyspnoea is defined by the American Thoracic Society as ‘a subjective experience of breathing discomfort that consists of qualitatively distinct sensations that vary in intensity’.^1 2^ Breathlessness accompanies many chronic conditions such as chronic obstructive pulmonary disease (COPD)^3^ and cancers of all types.^4 5^ It can have a profound effect on multiple aspects of quality of life.^6^ Patient assessment of breathlessness is fundamental to clinical management and research. There are growing calls for more routine assessment of breathlessness and a better understanding of the variation in reporting shown across groups of individuals.^7^ A range of tools are available such as the unidimensional Numerical Rating Scale (NRS) and the broader Multidimensional Dyspnea Profile (MDP),^8^ which assesses different domains of dyspnoea including emotional response.

The NRS, commonly used for self-reporting of subjective conditions, consists of a scale numbered 0–10, usually arranged vertically, anchored by a descriptive statement at each end and accompanied by a rating question^9^ (see online supplementary file: appendix A). A higher score represents greater symptom severity. The NRS was initially developed for pain assessment, where it is recommended,^10 11^ but has been adapted and recommended for breathlessness.^9 12 13^ In breathlessness, it is typically anchored with 0: ‘Not breathless at all’ and 10: ‘Breathlessness as bad as you can imagine’. A variety of questions are asked, most commonly: ‘What is the worst your breathlessness has been over the last 24 hours?’ (NRS-Worst), ‘How has your breathlessness been over the last 24 hours on average?’ (NRS-Average) and ‘How is your breathlessness now?’(NRS-Now).

10.1136/bmjresp-2017-000235.supp1 Supplementary file 1

One criticism of the NRS is that it has not been sufficiently evaluated.^9^ In COPD, validation is based on comparison with visual analogue scale (VAS) ratings.^14^ In patients with cancer, the most cited study surveyed just 31 patients but, despite this, it showed that the precise rating question asked is important: NRS-Worst requires a smaller sample size to detect a change in breathlessness than NRS-Average, and both require less than VAS equivalents.^15^

Recent studies using the NRS to assess breathlessness, in line with current recommendations,^9 12 13 16^ noted a subset of participants who, paradoxically, rated NRS-Average as worse than NRS-Worst.

The ‘Peak/End’ rule is a leading psychological theory that offers an explanation for the now-established differences between perception of an experience as it takes place (moment-based reporting) and subsequent retrospective assessment of the same experience (memory-based reporting).^17^ Since NRS-Average relies on memory-based reporting, the ‘Peak/End’ rule is potentially relevant. Broadly, it states that a when recalling an event—whether positive or negative—only the ‘Peak’ (the most extreme experience) and the ‘End’^18^ are taken into account while the duration of the event is overlooked.

## Methods

This paper seeks to highlight the existence of a subpopulation who give paradoxical averages using the NRS, to characterise this group and to investigate the explanatory relevance of the ‘Peak/End’ rule.

Data were collected within mixed method face-to-face interviews for three studies: the Living with Breathlessness Study (LwB)^19^ and the two subprotocols of the Breathlessness Intervention Service (BIS) phase III randomised controlled trial (RCT).^20 21^ LwB recruited a population-based sample of well-characterised patients with advanced COPD and their carers to a mixed method interview study seeking to improve care and support. The BIS phase III RCT was a mixed method pragmatic fast-track single-blinded RCT to evaluate a palliative care breathlessness intervention involving two subprotocols: one for patients and carers living with malignant conditions and one for non-malignant conditions.^22 23^

All three studies used NRS-Average, NRS-Worst and the Chronic Respiratory Questionnaire (CRQ; self-reported^24^ for LwB and interviewer administered^25^ for BIS). Both patients and carers participated; carers assessed patient breathlessness.

Key variables from the three datasets were pooled, and cases where participants reported a paradoxical average were identified. Cases and non-cases were compared with age and sex (patients and carers) and CRQ physical and emotional domains (patients only). Fisher’s exact test was used for categorical variables, and the Mann-Whitney U test was used for continuous variables.

Interview transcripts of the NRS administration were available for the LwB Study only. From these, extracts relating to NRS administration were reviewed for those cases that gave paradoxical averages. These brief extracts were reviewed to identify potential explanations for the paradoxical averages both in terms of verbalised participant thought processes and the way in which the scales were administered by the interviewers, for example, verbal explanations given to participants.

An additional analysis was conducted to explore the relevance of the Peak/End rule. This was restricted to the BIS datasets as they included NRS-Now. All NRS questions were asked in reference to the last 24 hours so this can be considered the duration of the experience for all data points. NRS-Worst was taken to represent the ‘Peak’ within those 24 hours, while NRS-Now represents the end of the experience. The mean of NRS-Worst and NRS-Now was calculated for each participant to represent their expected assessment following the Peak/End rule. These means were plotted against NRS-Average as a memory-based participant-generated assessment of the same period to assess distribution and potential correlation. Spearman’s rank correlation coefficient was calculated. Quantitative analyses were performed using SPSS (V.23).

## Results

Of 662 pooled patients and carers, 101 were excluded due to missing data for at least one variable and 45 were identified as paradoxical average cases. Table 1 summarises and compares the characteristics of cases and non-cases. Patients in the paradoxical average group had higher CRQ physical domain scores on average.

**Table 1:** Characteristics and comparison of cases and non-cases of paradoxical average

Participants	Non-cases of paradoxical average (average≤worst)	Cases of paradoxical average (average>worst)	p Value
Patients	n=350	n=21	
Patient age, median (IQR)	72 (65–79)	70 (64–78)	0.450
Patient sex, n (%)			
Male	201 (57)	9 (43)	0.257
Female	149 (43)	12 (57)	
CRQ* physical domain, median (IQR)	2.93 (2.11–3.78)	3.53 (2.78–3.89)	0.042
CRQ* emotional domain, median (IQR)	4.27 (3.36–5.27)	4.2 (3.73–4.48)	0.876

Carers	n=166	n=24	
Carer age, median (IQR)	68 (60–73)	67 (55–74)	0.815
Carer sex, n (%)			
Male	44 (27)	8 (33)	0.472
Female	122 (74)	16 (67)	

*Higher CRQ score reflects better health-related quality of life. LwB Study used the self-report version of the CRQ,^24^ whereas the two BIS RCT subprotocols used the interviewer administered version.^25^

BIS, Breathlessness Intervention Service; CRQ, Chronic Respiratory Questionnaire; LwB, Living with Breathlessness Study; RCT, randomised controlled trial.

A review of transcripts from cases identified within the LwB Study provided insights on aspects participants, and interviewers, found difficult. There was confusion about how to use the scale and the direction of scoring. After discussion, one patient commented ‘*I thought it was going up not down*’ concluding ‘*That question is crap [laugh] it’s badly written*’ (LwB208-003). The transcripts also suggest how participants might be assessing average. When asked about their worst breathlessness in the last 24 hours (NRS-Worst), one patient responded: ‘*not too bad at the minute because I haven’t been doing things*’ (LwB302-022). This suggests that, for this participant, current breathlessness influenced their retrospective assessment. In another case, there was discussion of specific events that occurred the day before that might have been linked to worst breathlessness; however, this finding was not universal.

Interviewer administration of the NRS varied. Sometimes interviewers sought to guide participants’ understanding of ‘average’, using phrases such as ‘*the good and the time it was the worst*’ (LwB302-022) and ‘*taking in the good and the bad’* (LwB400-013, LwB503-077 and LwB504-014). Furthermore, questions were asked in subtly, different ways with questions broken down into simpler parts:

I: … think about how you’ve been over the last 24 hours - so from 11 o’clock yesterday morning.

P: Okay.

I: What’s the worst that your breathlessness has been, on that scale? (LwB208-003)

Interviewers may also have had issues with the directionality of the scale. When one patient offered ‘*3 or 4*’ as an answer, the interviewer said they would record 4 ‘*because I’m going to go on the side of caution*’ (LwB503-077); this, however, represents a worse score. Another interviewer queried ratings of average and worst that were the same but accepted a higher revised estimate for average.

**Figure 1:**
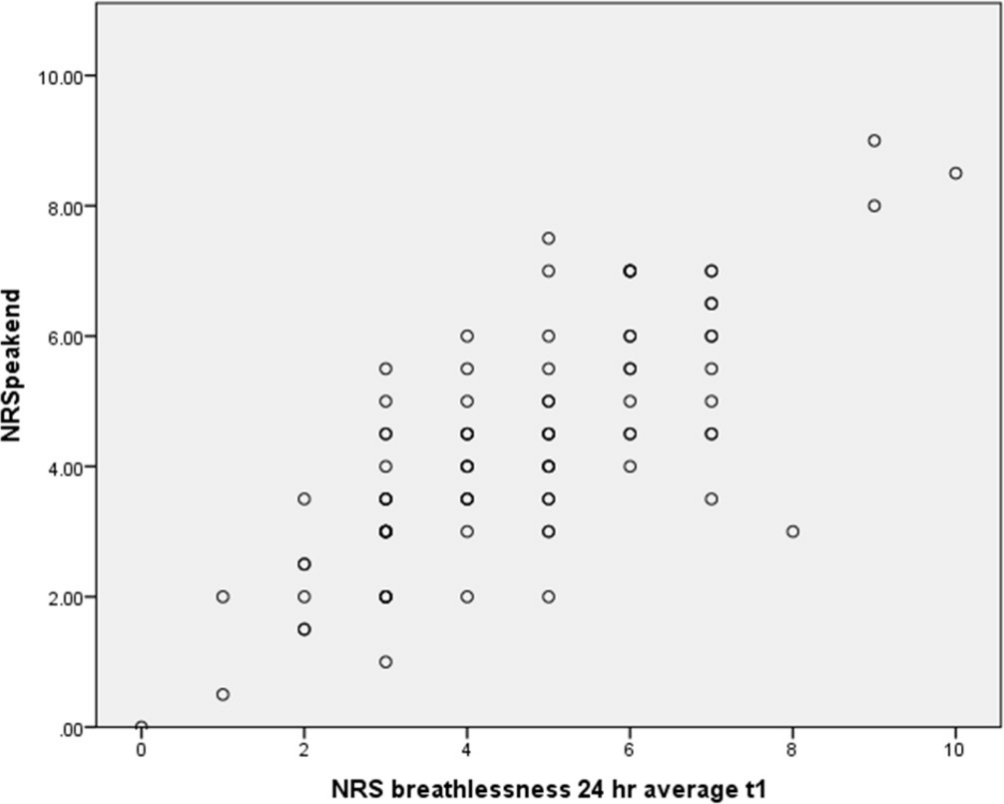
Predicted peak/end assessment against reported average breathlessness. NRS, Numerical Rating Scale.

Figure 1 presents the findings of the Peak/End rule analysis conducted on the BIS datasets. The monotonic association and high positive correlation (Spearman’s rho=0.756, p<0.001) shows a relationship between participant memory-based reporting and responses as predicted by the Peak/End rule. This strongly suggests that the Peak/End rule applies in this context and therefore that average breathlessness reporting is influenced by current breathlessness at the time of asking.

## Discussion

We identified a group of respondents who reported their average breathlessness as worse than their worst breathlessness in the past 24 hours. This was not just a patient-reporting problem but also one for some carers.

- Patients in this group had a higher CRQ physical domain score.
- Participants in this study have a high median age, while the NRS is shown to be more reliable in younger participants.
- Current breathlessness affected reporting of average breathlessness indicating the Peak/End rule applied.
- There were administration difficulties in using the scale in terms of directionality, interviewer variability and question structuring.

The existence of this group raises questions for the validity and utility of the NRS for symptom assessment without clear guidance. While the population we identified is small, it represents only those cases where confusion surrounding the scale resulted in paradoxical answers; the implication is that there may have been others who struggled with the scale but whose answers appeared valid. There are several possible explanations for our findings.

### The NRS itself

There is little standardisation in how the NRS is used, in terms of both its design and administration. In terms of design, it is reassuring that, for VAS scales, orientation appears not to matter for scales increasing left to right or up the page,^26^ and the NRS has been shown to be more reliable with patients younger than those in our samples when rating ‘peak’ breathlessness.^16^

In terms of administration, the NRS has been found to have the best response rate among established tools (even with over half returned incomplete in one study)^27^ and is recommended for clinical use because it is preferred by patients^28^; however, it is possible that these tools place an unexpected burden on patients. NRS questions are more complex than they at first appear. For example, ‘What is the worst your breathlessness has been over the last 24 hours?’ is not always asked using this precise form of words but rather split so that the patient is first directed to think about the last 24 hours and then about their worst breathlessness during that period. It could be argued that the first part evokes a similar intuitive construct to asking ‘average’ or ‘bother’ and subsequently asking worst represents changing the participant’s focus. Papers rarely report how the scale is introduced to participants. In the studies used for our analysis, a version of the NRS worsening down the page was used for consistency with the layouts of the Medical Research Council dyspnoea scale and Modified Borg Scale, which participants also completed; however, the effect of directionality on responses, especially those that were revised on questioning, is a topic for further research.

### Assessment of breathlessness at worst

Rating breathlessness at worst might seem the more intuitive of the two NRS questions administered (indeed, it has the lowest within-subject variability compared with the other questions^13^). It should be re-emphasised that a timeframe of the last 24 hours was used since reporting breathlessness has been shown to be consistent over the short timeframe of an emergency department visit but systematically biased when retested 4–6 weeks later when using the MDP.^29^ While there is a natural parallel to worst breathlessness, hospital admission has the strength of being a predefined event, and we have argued that patients may not be thinking of their breathlessness ‘at worst’ when answering about worst breathlessness.

One strategy to reduce errors may be to direct participants to think of and describe the worst experience in the last 24 hours and then rate it while looking at a scale show card. Examination of transcripts suggests that in some cases interviewers guided thinking in this way. This is akin to cognitive interviewing or think-aloud approaches^30^ and may help ensure the worst moment is not overlooked. Any attempt at using such techniques should still be standardised and reported as far as possible.

### Assessment of breathlessness on average

In asking participants to report averages, we are making assumptions that participants:

- understand the concept of average
- can compute an average that bears faithful relation to their experience
- and that assessment is repeatable and not influenced by factors relating to the asking of the question.

Wilcock *et al* 15 reported that asking patients to assess their ‘bother over the past 24 hours’ gave a very similar mean result to asking ‘average breathlessness over past 24 hours’. While this does not explicitly support the validity of the first assumption, the similarity in the means suggests the same construct is being assessed in both. Regardless of whether the term ‘average’ is understood in a mathematical sense, there seems to be an intuitive way in which participants interpret it and weigh a period of experience. If the same concept is assessed regardless, then it is irrelevant whether the concept of average is understood and furthermore this is outweighed by the known problems with ratings of past experience.

The application of the ‘Peak/End’ rule demonstrates the fallibility of memory-based reporting suggesting NRS-Average relies on the Peak and End, rather than reflecting a true average. Thus, what is being measured may not be what researchers or clinicians intend. This finding builds on research showing the validity of the ‘Peak/End’ rule in simulated breathlessness.^31^ Furthermore, while the rule may be time limited, the last 24 hours addressed in our study are well within the suggested boundary of 3 weeks.^32^ A corollary of the Peak/End rule is that experiences that are more variable are likely to be rated as more extreme overall since the ‘Peak’ is likely to be higher. This has been shown in patients with chronic pain.^33^

The ‘End’ is of interest as it is reasonable to assert that current breathlessness may act as a surrogate for this and is also widely measured. The construct validity of average breathlessness is therefore questionable. The effect of current breathlessness may be less important in a research setting as there may be greater consistency and control over the timing of assessments: in the studies used here, all respondents had been sitting for at least 15 minutes. A busy clinical environment is likely to be more variable. In outpatients, clinicians could reasonably usually avoid asking ‘now’ as this is likely context dependent and needs to be tightly constrained in reference to rest or exertion^34^; they may instead use worst or average, when they ask at all, but our findings suggest that when using NRS-Average, breathlessness ‘now’ may still play a role. A further area of investigation might relate to other temporal constructs used for breathlessness assessment such as ‘usual’,^14^ although the latter may still suffer from the same Peak/End rule issue.

### Assessing both

Asking multiple questions may compound confusion, especially when some questions appear to have multiple parts. The order in which questions are asked is not standardised, but order may affect answers given by directing participants to think of ‘worst’ or ‘current’ as benchmarks. Conversely, one could argue that asking more questions allows participants to become more aware of the nuance of the questions.

### Study limitations

Educational level and cognitive function (eg, due to suboptimal oxygen levels) may play a role in how participants answer rating questions; however, these data were only available for the LwB Study making the sample too small to address this hypothesis.

Furthermore, NRS administration data were only available as transcriptions; thus, it was not always possible to tell the extent to which interviewers used the provided NRS show cards. We cannot, therefore, comment on the extent to which show cards may help.

### Recommendations

The findings of this study suggest care should be employed in using any scale or tool; even apparently simple questions and response scales can be more complex to process than they appear. Responses can be influenced by many factors, and for this reason, we would recommend:

- establishment of the optimal order of questions where multiple questions are used
- standardisation of NRS administration and order where multiple questions are used
- reporting that standard rules and design have been used, or provision of a copy of the scale used
- further research into the effect of current breathlessness and situation on retrospective reporting (eg, by asking average breathlessness at the beginning and end of clinic consultations)
- a think-aloud study exploring thought processes participants go through when using the NRS to optimise its design.

## Conclusion

Breathlessness is fundamental to quality of life and so to clinical practice; it is increasingly recognised as an important predictor of outcome making routine assessment in everyone desirable.^7^ Efforts to improve clinical awareness and measurement of breathlessness have led to calls to recognise a clinical syndrome of chronic breathlessness.^35^ To capitalise on increased clinical awareness validity, standardisation and transparency in breathlessness measurement are essential. The NRS is a validated and highly recommended scale for breathlessness measurement, but this paper highlights that it requires care in choice of design and use. The application of the Peak/End rule demonstrates fallibility of NRS-Average as a construct that has implications for its utility.
