# Supplementary material for: Are within-person Numerical Rating Scale (NRS) ratings of breathlessness ‘on average’ valid in advanced disease for patients and for patients’ informal carers?
Source: BMJ Open Respir Res. 2017 Oct 11;4(1):e000235. doi: 10.1136/bmjresp-2017-000235 (PMC5652535; doi:10.1136/bmjresp-2017-000235)
Supplement: Supplementary file 1 [file bmjresp-2017-000235supp001.pdf]

Appendix A

1) What is the worst your breathlessness has been over the last 24 hours?

Please pick **one** number.

0 – Not breathless at all

1

2

3

4

5

6

7

8

9

10 – Breathlessness as bad as you can imagine

2) How has your breathlessness been over the last 24 hours on average?

Please pick **one** number.

0 – Not breathless at all

1

2

3

4

5

6

7

8

9

10 – Breathlessness as bad as you can imagine
